# RIP3 Inhibition ameliorates chronic constriction injury-induced neuropathic pain by suppressing JNK signaling

**DOI:** 10.18632/aging.203691

**Published:** 2021-11-12

**Authors:** Na He, Yu-Juan Qu, Dan-Yang Li, Shou-Wei Yue

**Affiliations:** 1 Rehabilitation Center, Qilu Hospital, Cheeloo College of Medicine, Shandong University, Jinan, China; 2 Institute of Rehabilitation Engineering, University of Health and Rehabilitation Sciences, Qingdao, China

**Keywords:** neuropathic pain, serine-threonine kinases receptor-interacting protein 3 (RIP3), c-Jun N-terminal kinase (JNK), sinomenine, neuroinflammation

## Abstract

Neuroinflammation is a major contributor to neuropathic pain. Receptor interacting serine/threonine kinase 3 (RIP3) senses cellular stress, promotes inflammatory responses and activates c-Jun N-terminal kinase (JNK) signaling. Here, we assessed the involvement of RIP3-induced JNK signaling in chronic constriction injury (CCI)-induced neuropathic pain. We found that RIP3 inhibitors (GSK’872) and JNK inhibitors (SP600125) not only alleviated the radiant heat response and mechanical allodynia in CCI rats, but also reduced inflammatory factor levels in the lumbar spinal cord. CCI surgery induced RIP3 mRNA and protein expression in the spinal cord. GSK’872 treatment after CCI surgery reduced RIP3 and phosphorylated (p)-JNK expression in the spinal cord, whereas SP600125 treatment after CCI surgery had almost no effect on RIP3. Sinomenine treatment reduced RIP3, p-JNK and c-Fos levels in the spinal cords of CCI rats. These data demonstrated that RIP3 inhibition (particularly via sinomenine treatment) alleviates neuropathic pain by suppressing JNK signaling. RIP3 could thus be a new treatment target in patients with neuropathic pain.

## INTRODUCTION

Lesions of the nervous system can lead to neuropathic pain (NP), a severe disease in which generally harmless stimuli induce hyperalgesia and spontaneous pain. NP can persist for months to years [1–3], causing considerable suffering to the affected patients and placing a substantial economic burden on individuals and countries [4, 5]. Analgesics such as tricyclic anti-depressants, non-steroidal anti-inflammatory drugs and opioids relieve symptoms by inhibiting neuronal activity, but their efficacy in hampering the progression of NP is not satisfactory [6–8]. In addition, these drugs have limited clinical applications due to their severe drug toxicity and dose-limiting side effects [9]. Thus, NP remains a major public health problem, and there is an urgent need to clarify its mechanism and develop better treatments.

The known pathogenic mechanism of NP is extremely complex, involving central and peripheral neuronal sensitization and neuroinflammation induced by inflammatory mediators [10]. Numerous studies have revealed that neuroinflammation contributes to the onset and continuation of NP by promoting the infiltration of immune cells, the activation of glial cells and the release of inflammatory mediators in the nervous system [10–14]. In addition, necrosis can induce the release of cellular constituents, thus stimulating or aggravating pain-related inflammation through various signaling mechanisms [15, 16]. Receptor interacting serine/threonine kinase 3 (RIP3) is known to be involved in necrosis [17], in addition to sensing cellular stress and inducing inflammatory responses; however, few studies have examined its participation in NP [18, 19].

Bennett and Xie proposed that chronic constriction injury (CCI) of the sciatic nerve could be used as a model of NP [20]. By applying this model, researchers have discovered various molecules that contribute to NP [21], including members of the mitogen-activated protein kinase (MAPK) pathway. For example, the activation of MAPK was found to induce the synthesis of tumor necrosis factor alpha (TNF-α) and promote mechanical tenderness after CCI. On the other hand, intrathecal lidocaine was reported to reduce tactile tenderness after CCI by suppressing the MAPK pathway in activated microglia [22–24]. C-Jun N-terminal kinase (JNK), a component of the MAPK signaling pathway, can be triggered by a variety of stress stimuli, and ultimately upregulates proinflammatory mediators that promote NP [25]. RIP3 has been reported to induce the JNK signaling pathway [26], but it is not clear whether this interaction contributes to NP.

Sinomenine, a natural biologically active ingredient extracted from the vine plant *Sinomenium acutum*, has been shown to reduce the erythrocyte sedimentation rate, antibody production, cytokine secretion and joint swelling in arthritis animal models [27]. Moreover, sinomenine has been reported to greatly alleviate various forms of NP by inhibiting neuroinflammation [28–30]. Repeated administration of sinomenine was found to improve the baseline pain threshold without producing tolerance [31, 32], and preconditioning with sinomenine was shown to delay morphine tolerance, suggesting that this compound has potential analgesic effects with long-term application. Therefore, we sought to determine the protective mechanism of sinomenine in NP. We first assessed the effects of RIP3 on the JNK signaling pathway in CCI-induced NP, and then investigated the influence of sinomenine on RIP3 in NP.

## RESULTS

### RIP3 inhibitors and JNK inhibitors alleviate the radiant heat response and mechanical allodynia in CCI rats

**Figure 1:**
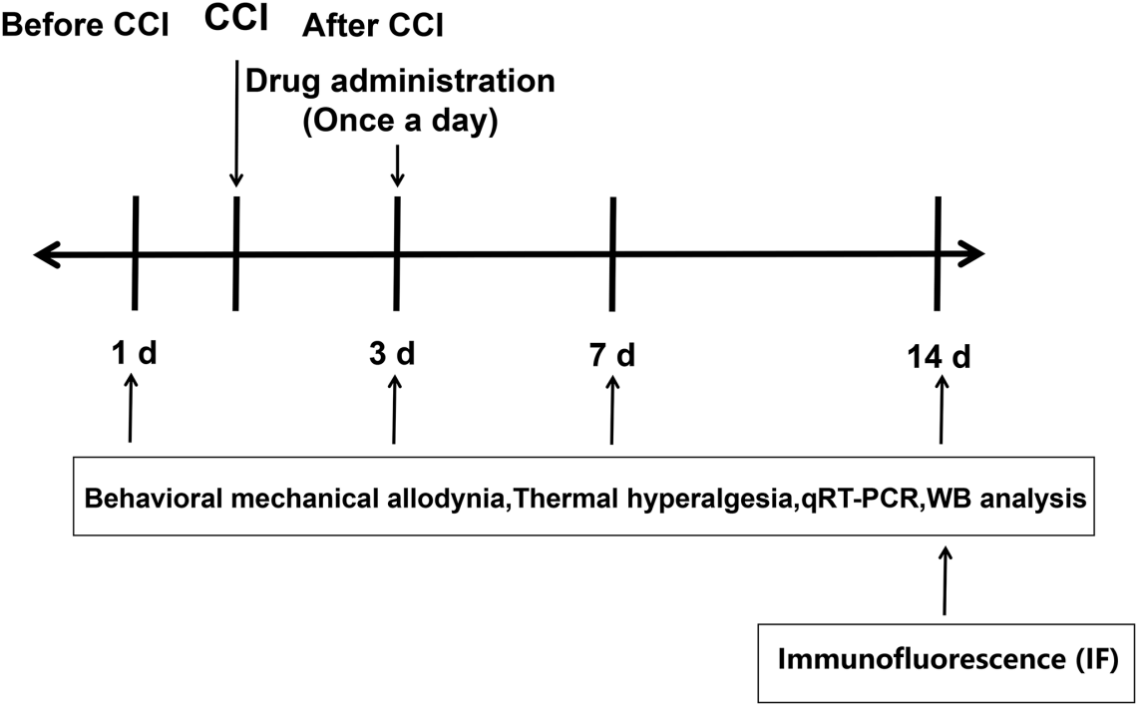
Experimental design.

**Figure 2:**
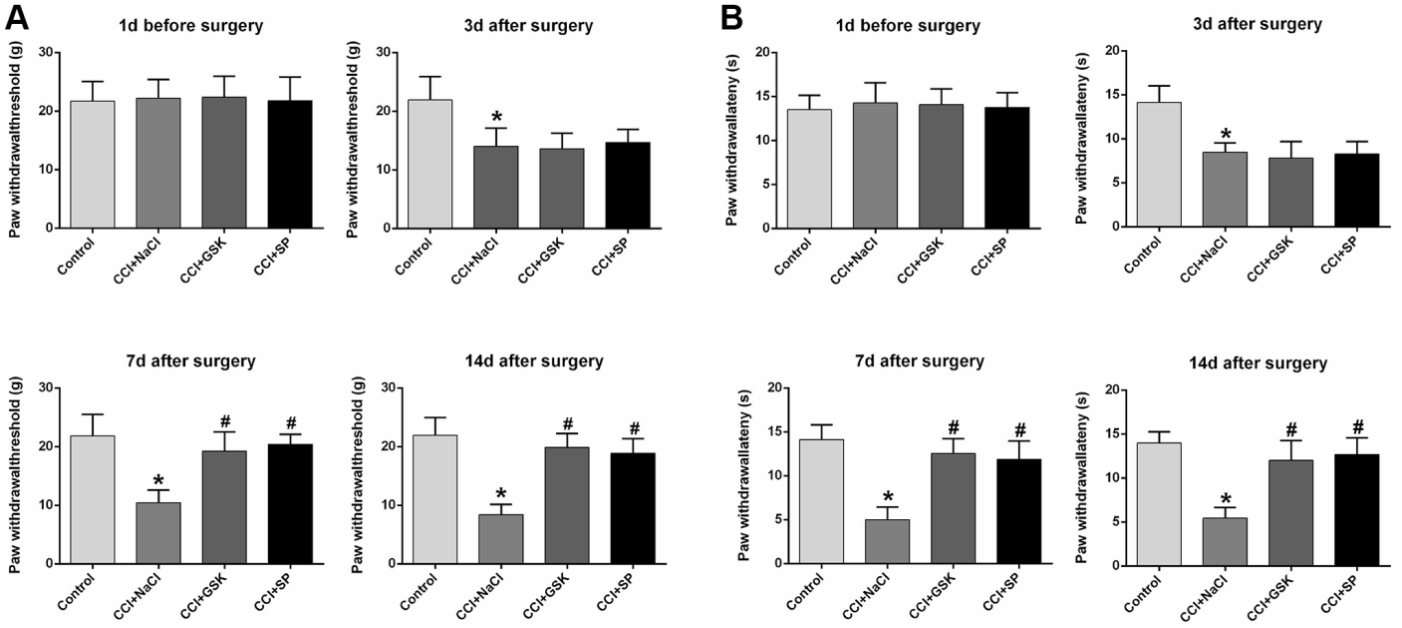
**The influences of RIP3 inhibitor and JNK inhibitor on pain behaviors of CCI rats.** (**A**) The behavioral mechanical allodynia at day 1 before CCI and at days 3, 7, and 14 after CCI. (**B**) The thermal hyperalgesia at day 1 before CCI and at days 3, 7, and 14 after CCI. Data are shown as mean SD (*n* = 8). ^*^P < 0.05, in contrast with the control group, ^#^*P* < 0.05, in contrast with CCI + NaCl group.

The experimental design was shown in Figure 1. To assess the involvement of RIP3 and JNK in CCI-induced NP, we randomly divided rats into four groups: rats subjected to a sham CCI operation (the control group); CCI rats injected with saline (the CCI+NaCl group); CCI rats injected with the RIP3 inhibitor GSK’872 (the CCI+GSK group); and CCI rats injected with the JNK inhibitor SP600125 (the CCI+SP group). Pain behavior was monitored at different stages (1 day before CCI, 3 days after CCI, 7 days after CCI and 14 days after CCI) based on the paw withdrawal latency (PWL) in response to radiant heat and the paw withdrawal threshold (PWT) in response to mechanical allodynia. The PWL and PWT values of CCI+NaCl rats were noticeably lower than those of control rats (*P* < 0.05). However, the PWL and PWT values 7 and 14 days after CCI were distinctly greater in the CCI+GSK and CCI+SP groups than in the CCI+NaCl group (*P* < 0.05; Figure 2A and 2B). Thus, RIP3 inhibitors and JNK inhibitors both alleviated nociceptive behaviors in CCI rats. These results suggested that RIP3 and JNK may contribute to the development of CCI-induced NP.

### RIP3 is involved in CCI-induced inflammatory responses

**Figure 3:**
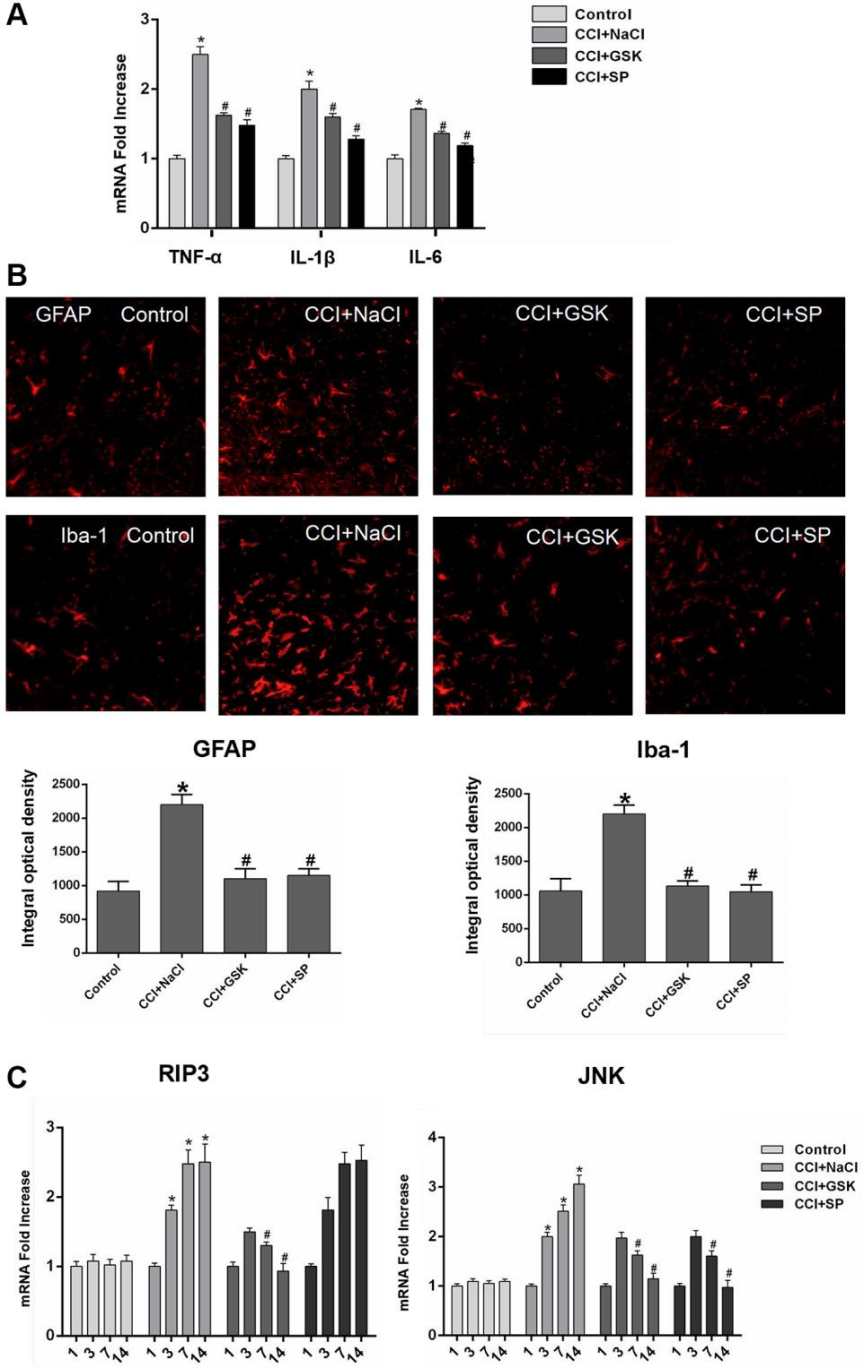
**The mRNA expressions of inflammatory cytokines, RIP3 and JNK in the lumbar spinal cords of CCI rats with qRT-PCR.** (**A**) The mRNA expressions of TNF-α, IL-1β and IL-6 in the lumbar spinal on 14th day after CCI. (**B**) The expression of GFAP and Iba-1 in lumbar spinal cord by immunofluorescence on 14 days after CCI. (**C**) The mRNA levels of RIP3 and JNK in the lumbar spinal on 1 day before CCI and 3, 7, and 14 days after CCI. Data are presented as mean SD (*n* = 8). ^*^P < 0.05, in contrast with the control group, ^#^*P* < 0.05, in contrast with the CCI + NaCl group.

Given that nerve inflammation is a major pathogenic factor in NP, we used quantitative real-time PCR (qRT-PCR) to analyze proinflammatory cytokine levels in the lumbar spinal cords of CCI rats 14 days after surgery. The mRNA levels of *TNF-α*, interleukin 6 (*IL-6*) and *IL-1β* in the lumbar spinal cord were greater in CCI+NaCl rats than in control rats (*P* < 0.05), further illustrating the successful establishment of the experimental model. However, the mRNA levels of these cytokines were noticeably reduced in the CCI+GSK and CCI+SP groups compared with the CCI+NaCl group (*P* < 0.05; Figure 3A).

In the central nervous system, microglia monitor the local environment to respond quickly to a wide range of inflammatory stimuli, while astrocytes function as immune response cells to produce a large number of inflammatory mediators [6, 33]. To evaluate the effects of RIP3 and JNK inhibitors on the density of glial cells and the activation of microglia and astrocytes *in vivo*, we stained for the astrocyte-specific marker glial fibrillary acidic protein (GFAP) and the microglia- specific marker ionized calcium-binding adaptor molecule 1 (IBA-1). Quantitative analyses indicated that the proportion of activated microglia and astrocytes increased after CCI but decreased significantly after GSK’872 or SP600125 treatment (*P* < 0.05; Figure 3B). These findings demonstrated that RIP3 and JNK participate in the inflammatory response induced by CCI.

### RIP3 and JNK expression in the spinal cords of CCI rats

**Figure 4:**
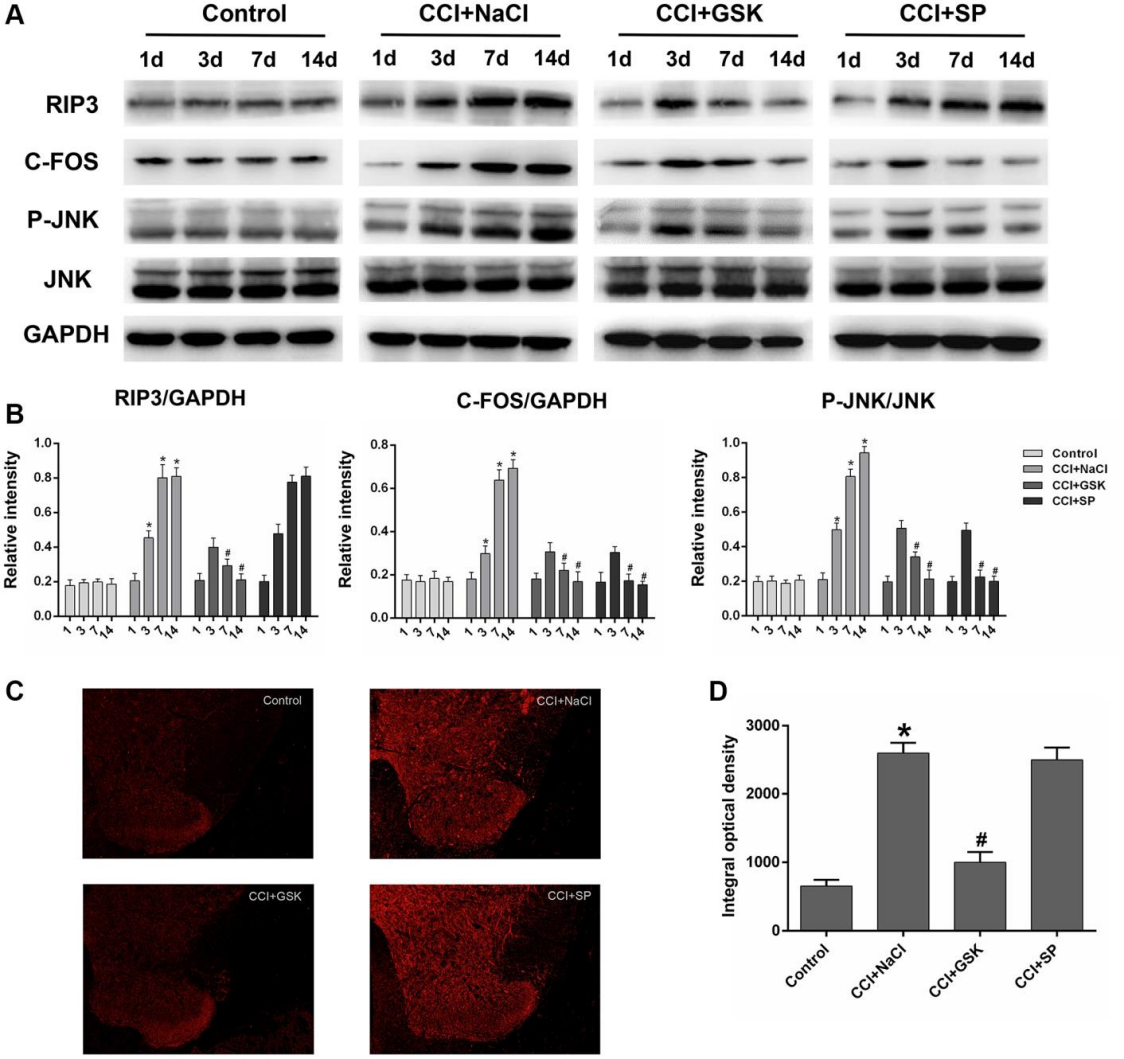
**The influences of RIP3 inhibitor and JNK inhibitor on the levels of RIP3 and p-JNK in the lumbar spinal cords of CCI rats.** (**A**) The levels of RIP3, p-JNK, JNK, c-Fos, and GAPDH in the lumbar spinal cords of rats on 1 day before CCI and 3, 7, and 14 days after CCI. (**B**) The ratio of RIP3, c-Fos to GAPDH and the ratio of p-JNK to JNK were analyzed. (**C**) The expression of RIP3 in lumbar spinal cord by immunofluorescence on 14 days after CCI. (**D**) Quantification of immunofluorescence area of (**C**) by Image J. Data are shown as mean SD (*n* = 8). ^*^P < 0.05, in contrast with the control group, ^#^*P* < 0.05, in contrast with the CCI + NaCl group.

Having determined that RIP3 and JNK were involved in pain-related behavioral changes and inflammatory responses in CCI rats, we next applied qRT-PCR and Western blotting to evaluate the expression of RIP3 and JNK in the lumbar spinal cord 1 day before and 3, 7 or 14 days after CCI. Since c-Fos expression has been reported to increase during NP [34]., we employed c-Fos as a marker of NP. On days 3, 7 and 14 after surgery, *RIP3* and *JNK* mRNA levels in the spinal cord were greater in the CCI+NaCl group than in the control group (*P* < 0.05; Figure 3C). Likewise, RIP3, phosphorylated (p)-JNK and c-Fos protein levels in the spinal cord were greater in the CCI+NaCl group than in the control group on days 3, 7 and 14 after surgery (*P* < 0.05; Figure 4A). On postoperative days 7 and 14, *RIP3* and *JNK* mRNA levels in the spinal cord gradually decreased in the CCI+GSK group compared with the CCI+NaCl group (*P* < 0.05). In the CCI+SP group, *JNK* mRNA levels in the spinal cord were also reduced on postoperative days 7 and 14; however, there was no evident alteration in *RIP3* mRNA levels (*P* < 0.05; Figure 3C). In addition, on days 7 and 14 after CCI surgery, RIP3, p-JNK and c-Fos protein levels in the spinal cord were noticeably lower in the CCI+GSK group than in the CCI+NaCl group (*P* < 0.05). On postoperative days 7 and 14, p-JNK and c-Fos protein levels in the spinal cord were distinctly lower in the CCI+SP group than in the CCI+NaCl group, but RIP3 protein levels exhibited little variation in the CCI+SP group (*P* < 0.05; Figure 4A and 4B). These data demonstrated that inhibiting RIP3 significantly suppressed JNK in the spinal cord.

To verify these results, we performed immunofluorescence assays to evaluate RIP3 expression in the spinal cord. RIP3 expression was noticeably greater in the CCI+NaCl group than in the control group, but was clearly lower in the CCI+GSK group than in the CCI+NaCl group (*P* < 0.05). Consistent with the above experimental results, RIP3 expression was not evidently altered in the CCI+SP group (*P* < 0.05; Figure 4C and 4D). These findings confirmed that RIP3 and JNK signaling promote the development of CCI-induced NP, with RIP3 functioning upstream of JNK.

### RIP3 induces neuronal damage by stimulating JNK signaling

**Figure 5:**
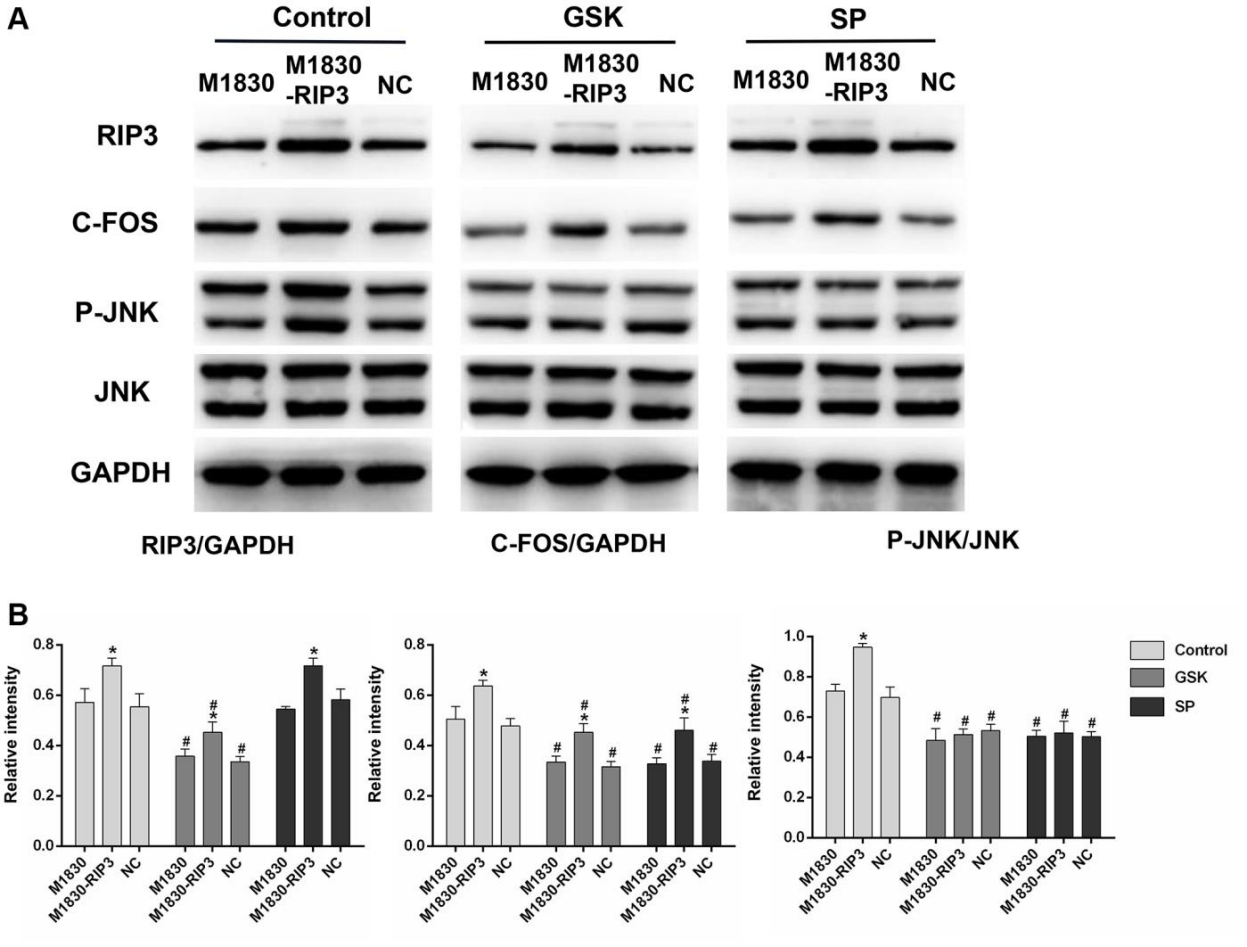
**The variation in the expression of RIP3, p-JNK, and c-Fos in RIP3-overexpressed astrocytes after treatment with RIP3 inhibitors and JNK inhibitors.** (**A**) The levels of RIP3, p-JNK, JNK, c-Fos and GAPDH in diverse groups of astrocytes. (**B**) The ratio of RIP3 or c-Fos to GAPDH and the ratio p-JNK to JNK were quantified with Image J. Data are shown as mean SD (*n* = 8). ^*^P < 0.05, in contrast with the M1830 cells within groups, ^#^*P* < 0.05, in contrast with the control group.

To further explore the functions of RIP3 and p-JNK, we transfected astrocytes with a *RIP3* overexpression plasmid or a negative control (NC) plasmid, and then treated these cells with GSK’872 or SP600125. GSK’872 distinctly weakened RIP3, p-JNK and c-Fos protein expression in astrocytes transfected with the *RIP3* plasmid or the NC plasmid; however, SP600125 reduced p-JNK and c-Fos levels without affecting RIP3 (*P* < 0.05; Figure 5A and 5B). These findings illustrated that RIP3 promotes neuronal damage by inducing JNK signaling.

**Figure 6:**
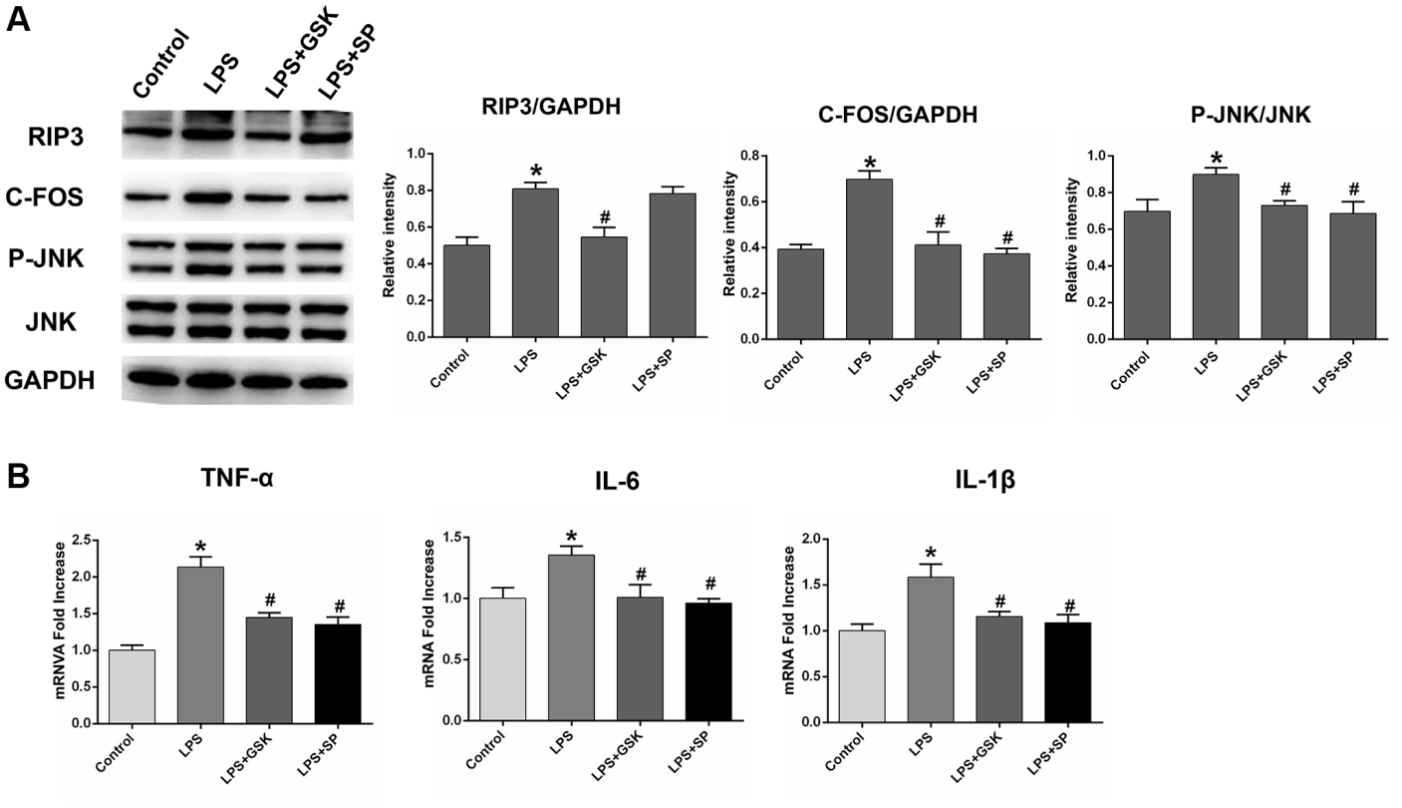
**The variation in the expression of RIP3, p-JNK, and c-Fos in LPS-stimulated astrocytes after treatment with RIP3 inhibitors and JNK inhibitors.** (**A**) The levels of RIP3, p-JNK, JNK, c-Fos and GAPDH in different groups of astrocytes. (**B**) The mRNA levels of TNF-α, IL-1β and IL-6 in different groups of astrocytes. Data are shown as mean SD (*n* = 8). ^*^P < 0.05, in contrast with control groups, ^#^*P* < 0.05, in contrast with the LPS group.

Lipopolysaccharide (LPS) is widely used in experimental studies of neuroinflammatory responses [35, 36]. To verify that RIP3 induces neuronal damage by promoting JNK signaling, we divided astrocytes into four groups: the control group, the LPS group, the LPS+GSK group and the LPS+SP group. RIP3, p-JNK and c-Fos protein levels were obviously greater in the LPS group than in the control group (*P* < 0.05). Following GSK’872 treatment, RIP3, p-JNK and c-Fos protein levels were reduced in LPS-treated cells (*P* < 0.05). After SP600125 treatment, p-JNK and c-Fos protein levels were reduced in LPS-treated cells, but RIP3 expression was not significantly altered (*P* < 0.05; Figure 6A).

We also examined inflammatory factor levels in the various groups of cells. *TNF-α*, *IL-1β* and *IL-6* mRNA levels were noticeably greater in the LPS group than in the control group. In the LPS+GSK and LPS+SP groups, *TNF-α*, *IL-1β* and *IL-6* mRNA levels were reduced, indicating that both GSK’872 and SP600125 reduced the inflammatory response induced by LPS (*P* < 0.05; Figure 6B). These results provided sufficient evidence that RIP3 induces neuronal damage by upregulating the JNK signaling pathway.

### Sinomenine exerts remarkable analgesic effects during NP by suppressing RIP3

**Figure 7:**
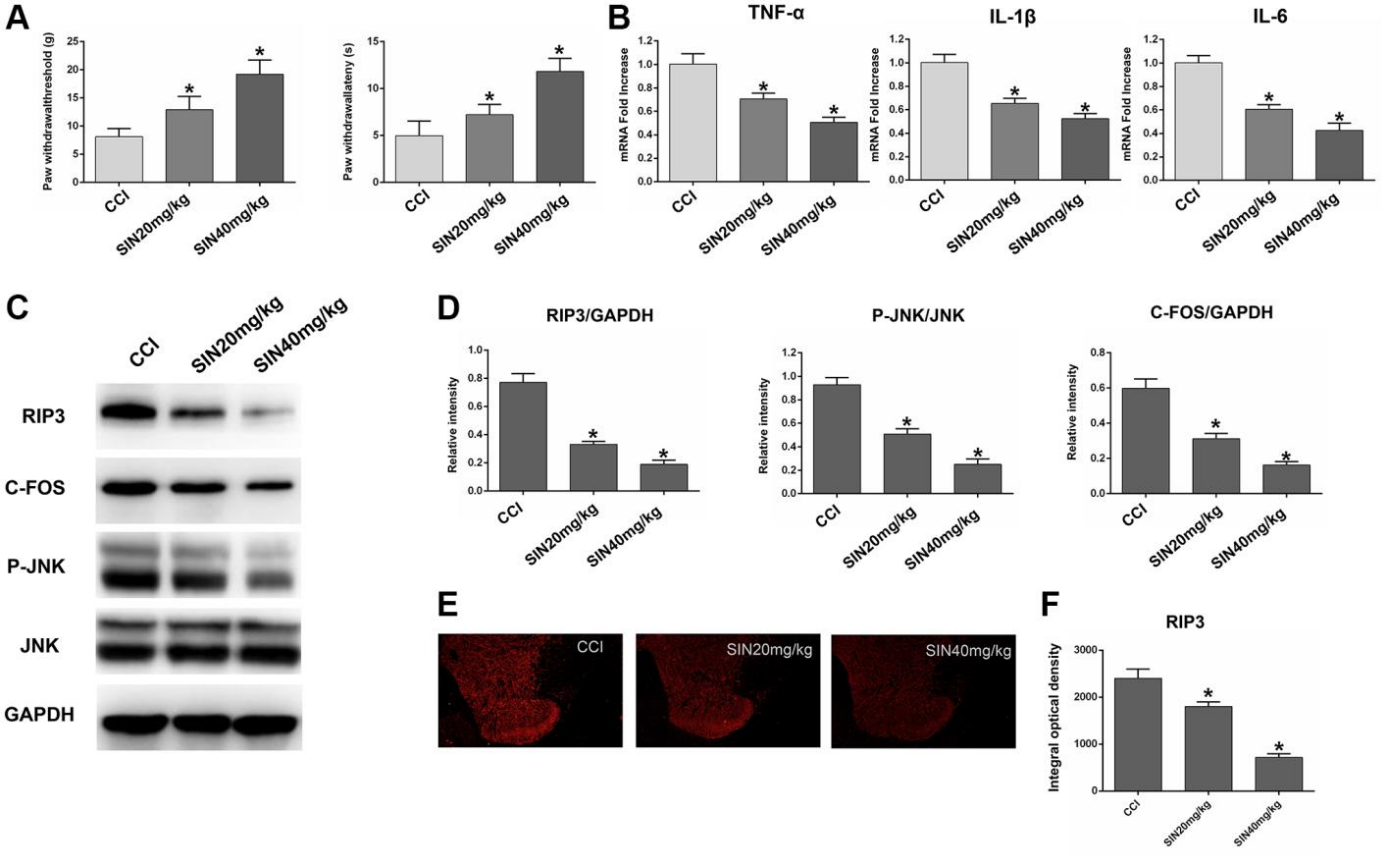
**Effects of sinomenine on CCI rats.** (**A**) The behavioral mechanical allodynia and thermal hyperalgesia of CCI rats treated with sinomenine at days 14 after operation. (**B**) The mRNA expressions of TNF-α, IL-1β and IL-6 in the lumbar spinal cords of rats treated with sinomenine via qRT-PCR at 14 days after operation. (**C**) The levels of RIP3, p-JNK, JNK, c-Fos, and GAPDH in the lumbar spinal cords of rats treated with sinomenine at 14 days after operation. (**D**) The ratio of RIP3, c-Fos to GAPDH and the ratio of p-JNK to JNK were analyzed. (**E**) The expression of RIP3 in lumbar spinal cord of rats treated with sinomenine by immunofluorescence on 14th day after operation. (**F**) Quantification of immunofluorescence area of (**C**) with Image J. Data are shown as mean SD (*n* = 8). ^*^P < 0.05, contrasted to the CCI group.

We next explored the protective effects of sinomenine during NP by using three groups of rats: the CCI group, the CCI+sinomenine (SN) 20 mg/kg group and the CCI+SN 40 mg/kg group. Since chronic neuronal pain (such as CCI-induced NP) requires repeated drug treatments due to its persistent nature, we assessed the effects of sinomenine on CCI-induced NP after two weeks of treatment. First, we investigated the influence of different doses of sinomenine on the radiant heat response and mechanical allodynia in CCI rats. As shown in Figure 7A, two-week sinomenine treatment dose-dependently increased the PWL and PWT values in CCI rats (*P* < 0.05).

Next, we used qRT-PCR to assess *TNF-α*, *IL-1β* and *IL-6* expression in the lumbar spinal cord. The mRNA levels of *TNF-α*, *IL-1β* and *IL-6* decreased dose-dependently in sinomenine-treated rats compared with CCI rats (*P* < 0.05; Figure 7B). These results demonstrated that sinomenine has significant therapeutic effects against NP.

Subsequently, we explored whether sinomenine exerted its analgesic effects during NP by altering RIP3 signaling. RIP3 and p-JNK protein levels in the spinal cord were reduced in sinomenine-treated CCI rats compared with CCI rats, with more marked reductions in the high-dose group (*P* < 0.05; Figure 7C and 7D). In addition, c-Fos levels in the spinal cord were lower in sinomenine-treated CCI rats than in CCI rats (*P* < 0.05). Immunofluorescence analyses also indicated that RIP3 levels in the spinal cord were lower in the CCI+SN 20 mg/kg and CCI+SN 40 mg/kg groups than in the CCI group (*P* < 0.05; Figure 7E and 7F). Consistent with the Western blotting data, the decline in RIP3 expression was more significant in the high-dose sinomenine-treated group (*P* < 0.05).

Lastly, to confirm the therapeutic action of sinomenine, we stained for NeuN, a specific marker of living neurons [37]. The results revealed that the number of active neurons in the spinal dorsal horn was lower in CCI rats than in control rats. However, the number of living neurons in the spinal dorsal horn was greater in CCI+SN rats than in CCI rats, illustrating that sinomenine inhibited CCI-induced neuronal death (*P* < 0.05; Supplementary Figure 1). These data strongly suggested that sinomenine exerts significant analgesic and therapeutic effects against NP by suppressing RIP3.

### DISCUSSION

Peripheral nervous system injuries due to trauma, disease or surgery can lead to NP, causing immense suffering to patients [38]. Excessive neuroinflammation has been shown to initiate and sustain NP. During the inflammatory response, the release of chemokines, lipid mediators and cytokines can stimulate and sensitize nociceptors, and these adverse effects may promote persistent NP [39]. RIP3 is a crucial regulator of inflammation [40–43]. Independently of mixed lineage kinase domain-like pseudokinase (MLKL) and necrotic cell death, RIP3 has been found to facilitate inflammatory responses involving the NLR family pyrin domain-containing 3 inflammasome and IL-1β [44]. Moreover, necrostatin-1 has been reported to ameliorate peripheral nerve injury-induced NP by inhibiting the RIP1/RIP3 pathway [45].

A variety of stress stimuli can trigger the JNK signaling pathway, which participates in the inflammatory response by regulating inflammatory cytokine and chemokine expression [46, 47]. JNK signaling is also an important contributor to chronic inflammatory pain, and may be continuously activated after nerve damage [24]. Importantly, previous studies have indicated that reducing RIP3-induced inflammation can meaningfully weaken JNK signaling; for instance, the inhibition of RIP3 by miR-325-3p was found to deactivate MAPK pathway members such as P38 and JNK, hence preserving neurons from damage due to oxygen-glucose deprivation/re-oxygenation [26]. While these findings revealed the relationship between RIP3 and JNK, it remained unclear whether RIP3-induced JNK signaling contributed to CCI-induced NP. In our research, we adopted CCI as an experimental model to study the involvement of RIP3 and JNK in NP.

The main pathological manifestations of peripheral nerve damage-induced chronic NP are allodynia and hyperalgesia in response to thermal and mechanical stimuli [48]. Thus, we assessed the PWT and PWL to evaluate whether GSK’872 or SP600125 treatment altered nociceptive behavior in CCI rats. The PWT and PWL values were higher in the CCI+GSK and CCI+SP groups than in the CCI+NaCl group, indicating that both of these treatments had effective anti-nociceptive properties in rats after CCI surgery. Neuroinflammation is another important pathogenic factor in NP [9], so we also used qRT-PCR to measure the levels of proinflammatory cytokines such as *TNF-α*, *IL-1β* and *IL-6* in the lumbar spinal cord. As expected, CCI surgery increased the levels of these cytokines, but GSK’872 and SP600125 reduced them by the 14th day after CCI surgery.

To determine the molecular mechanism through which RIP3 and JNK inhibitors reduced NP, we then evaluated RIP3 and JNK levels in the spinal cords of CCI rats. CCI surgery significantly induced RIP3 expression in the spinal cord, but GSK’872 treatment after CCI surgery reduced RIP3 and JNK levels. SP600125 treatment after CCI surgery reduced JNK expression but had little effect on RIP3 expression. The expression of c-Fos (a marker of NP [34]) was also upregulated in the spinal cord after CCI, similar to RIP3; however, c-Fos was downregulated after both GSK’872 and SP600125 treatment. Immunofluorescence experiments revealed the same RIP3 expression patterns as the Western blotting and qRT-PCR assays in the lumbar spinal cord. These data indicated that antagonizing RIP3 could prevent the activation of the JNK pathway and exert anti-nociceptive effects in a model of NP.

Astrocyte activation due to inflammation and peripheral nerve injury can maintain NP. Astrocytes not only produce high levels of inflammatory mediators as immune response cells, but also offer stable niches for signal transduction between neurons. Thus, analgesic strategies targeting astrocytes have been explored as advanced methods to treat NP [6, 49]. In this study, we used wild-type and *RIP3*-overexpressing astrocytes to verify the effects of GSK’872 and SP600125. The protein levels of RIP3, p-JNK and c-Fos increased visibly in *RIP3*-overexpressing astrocytes. GSK’872 reduced RIP3, p-JNK and c-Fos expression, while SP600125 only reduced p-JNK and c-Fos levels. These results further established that RIP3 promotes neuronal damage by upregulating JNK.

Numerous studies have shown that LPS administration triggers neuroinflammatory responses in astrocytes, thereby activating inflammatory pathways and inducing inflammatory cytokine secretion [50]. In our research, LPS treatment clearly upregulated RIP3, p-JNK and c-Fos in astrocytes. GSK’872 treatment reduced RIP3, p-JNK and c-Fos levels in LPS-stimulated cells, whereas SP600125 treatment only reduced p-JNK and c-Fos expression. Both GSK’872 and SP600125 reduced LPS-induced inflammatory cytokine production and diminished the number of activated microglia and astrocytes. These results strengthened the evidence that RIP3 induces neuronal damage by stimulating JNK.

Sinomenine is an effective anti-inflammatory and analgesic substance in both the peripheral nervous system and the central nervous system, with the ability to balance neuro-immune interactions and inhibit central sensitization under neuroinflammation during chronic pain [27]. Sinomenine can block the peripheral sodium channel, reduce glutamate levels, activate the γ-aminobutyric acid A receptor and directly inhibit neuronal firing, so it has great potential to alleviate NP in different clinical environments [51]. In addition, sinomenine can inhibit nuclear factor κB and P38 activity, thus relieving neuroinflammation, ameliorating the external environment of neurons and exerting analgesic effects [52]. Sinomenine may be a multi-target drug, as its pharmacological properties are diverse and its specific target has not yet been discovered [53].

Our study indicated that sinomenine treatment dose-dependently increased the PWL and PWT values of CCI rats, illustrating that sinomenine can significantly relieve thermal hyperalgesia and mechanical allodynia. In addition, sinomenine dose-dependently reduced *TNF-α*, *IL-1β* and *IL-6* mRNA levels in the lumbar spinal cord, demonstrating that sinomenine suppressed the inflammatory response in CCI rats. RIP3, p-JNK and c-Fos levels in the spinal cord also decreased in CCI rats treated with sinomenine, and these effects were more significant in the high-dose group. Moreover, sinomenine increased the number of live neurons in the spinal dorsal horn of CCI rats, indicating that sinomenine can alleviate CCI-induced neuronal death. These results suggested that sinomenine alleviates NP by suppressing RIP3-induced JNK signaling. Although its mechanism and applicability need to be further verified, we believe that sinomenine has great clinical potential for the treatment of NP.

In conclusion, our data demonstrated that RIP3 expression and JNK signaling were activated in CCI-induced NP. RIP3 inhibitors (GSK’872) and JNK inhibitors (SP600125) effectively relieved the pain caused by CCI. GSK’872 was found to suppress the JNK pathway after CCI, indicating that RIP3 inhibitors ameliorate NP by inhibiting JNK signaling and the associated inflammation. Sinomenine also alleviated NP by diminishing RIP3-induced JNK signaling. Therefore, RIP3 could be considered as a new treatment target in NP.

## MATERIALS AND METHODS

### Materials

The following antibodies were purchased from Abcam Trading Company (Abcam, USA): RIP3 (1:1000), p-JNK (1:1000), JNK (1:1000) and c-Fos (1:1000). LPS was acquired from Sigma (St. Louis, MO, USA). The *RIP3* cDNA plasmid and the NC plasmid were synthesized by Shanghai Biosune Company. Sinomenine, GSK’872 and SP600125 were purchased from Shanghai MedChemExpress, LLC.

### Animals

Adult male Sprague-Dawley rats (200–250 g) from Beijing Vital River Laboratory Animal Technology Co., Ltd. were maintained at a room temperature of 22 ± 2°C with free access to food and water. This animal protocol was reviewed and approved by the Animal Care and Use Committee of Shandong University. We used the smallest number of rats statistically possible for each part of our research.

### Surgery and drugs treatment

#### Surgery and drugs treatment

For the CCI operation, we first anesthetized the rats via intraperitoneal injection of chloral hydrate (40 mg/kg). The biceps femoris muscle was obtusely dissected and the lateral common sciatic nerve was exposed. Proximal to the sciatic trifurcation, approximately 6 mm of nerve was detached from the adhesive tissues, and four ligations were loosely bound around it at intervals of approximately 1 mm, such that the affected nerve length was 4.5 mm. The rats in the control group were subjected to the same operation without the sciatic nerve ligation. The rats in the CCI+NaCl group were intraperitoneally injected with 20 μL of normal saline daily on postoperative days 3 to 14. The rats in the CCI+GSK group were intrathecally injected with GSK’872 (an inhibitor of RIP3, 25 μL/kg) daily on postoperative days 3 to 14. The rats in the CCI+SP group were intrathecally injected with SP600125 (an inhibitor of JNK, 10 μg/kg) for the same duration. We injected into the subarachnoid space between the L5 and L6 vertebrae, and verified the correct position by the appearance of a tail flick.

For the sinomenine treatment, sinomenine was first dissolved in dimethyl sulfoxide (Sigma-Aldrich) and then diluted in saline. Sinomenine was orally administered to the rats daily for 14 days at a dose of either 20 mg/kg (CCI+SN 20 mg/kg group) or 40 mg/kg (CCI+SN 40 mg/kg group). After 14 days, samples were collected for the experimental study.

### Nociceptive behavior

Prior to the baseline behavioral analysis, the rats were acclimated to the test environment for at least two days. A case was prepared on a high metal mesh floor, and the rats were placed in the case and allowed to adjust to the test environment for 30 minutes. Von Frey testing was used to assess mechanical sensitivity. For the PWT test, a series of von Frey hairs were applied with sufficient force on the lateral plantar surface of the hind paw. Immediate retraction of the paw was considered to be a positive reaction. The experiment was repeated three times at intervals of at least five minutes, and the average was registered. Only animals with a normal gait without foot deformities were used for the subsequent experiments.

A BME-410C thermal analgesia tester (CAMS) was employed to determine the PWL of the rats. The rats were randomly placed in a laboratory chamber to acclimate for 30 minutes before the experiment. The thermal analgesia tester was adjusted to generate a 10- to 14-second latency period for paw release in the control rats. Radiating light was shone through a glass plate directly into the center of the rat’s hind paw, and the PWL was mechanically measured when the rat licked or lifted the paw. To protect the rear claw tissue, a cut-off time of 20 seconds was set. Each hind paw was stimulated three times at intervals of five minutes, and the mean withdrawal latency was registered.

### Western blotting

Treated cells and lumbar spinal cord tissues were collected. Proteins were isolated, separated on sodium dodecyl sulfate polyacrylamide gels and transferred to polyvinylidene difluoride membranes. The blots were incubated with antibodies against RIP3, JNK, p-JNK, c-Fos and GAPDH (the loading control). The blots were observed using an enhanced chemiluminescence system (Millipore, Beijing, China). Image J software (NIH, Bethesda, MD, USA) was used to analyze the signal intensity.

### qRT-PCR

Trizol reagent (Invitrogen, Carlsbad, CA, USA) was used to extract total RNA from lumbar spinal cord samples and cells. PCR analysis was carried out in accordance with the manufacturer’s protocol (Toyobo, Japan). Melting curves were generated at the end of the cycles to verify the absence of non-specific products. The 2^−ΔΔCT^ method was used to quantify mRNA levels. *GAPDH* was applied as an endogenous control to normalize differences in mRNA detection. The following primers were used: RIP3 (forward, 5′-TCGTGGGCTCTGAAGAACTG-3′; reverse, 5′-ACCATAGCCTTCACCTCCCT-3′); GAPDH (forward, 5′-TCTCTGCTCCTCCCTGTTCT-3′; reverse, 5′-ATCCGTTCACACCGACCTTC-3′); TNF-α (forward, 5′-ATGGGCTCCCTCTCATCAGT-3′; reverse, 5′-GCTTGGTGGTTTGCTACGAC-3′); IL-1β (forward, 5′-GGGATGATGACGACCTGCTA-3′; reverse, 5′-ACAGCACGAGGCATTTTTGT-3′); IL-6 (forward, 5′-TTTCTCTCCGCAAGAGACTTCC-3′; reverse, 5′-TGTGGGTGGTATCCTCTGTGA-3′); JNK (forward, 5′-ACAGACCTAAGTACGCTGGC-3′; reverse, 5′-ACCAGACGTTGATGTACGGG-3′).

### Immunofluorescence staining

On the 14th postoperative day, dorsal root ganglion cross-sections (surgical side, 4 mm) were prepared from the different groups. The sections were fixed with 4% paraformaldehyde, permeated with 0.1% Triton X-100, and then blocked with 10% normal serum at room temperature for two hours to prevent non-specific staining. Subsequently, the sections were treated with primary antibodies against RIP3 at 4°C overnight. The sections were washed twice for five minutes with phosphate-buffered saline, and then were treated with fluorescein isothiocyanate-conjugated secondary antibodies at 37°C in the dark for one hour. Finally, the prepared sections were washed with phosphate-buffered saline for five minutes, counterstained with 4’,6-diamidino-2-phenylindole (DAPI) and imaged using a confocal microscope.

### Cell culture and treatments

The mouse spinal cord astrocyte cell line M1830 was obtained from Procell Life Science and Technology Co., Ltd. The cells were incubated in Dulbecco’s modified Eagle’s medium supplemented with 10% endotoxin-free fetal bovine serum, and were cultivated in an atmosphere of 95% O_2_ and 5% CO_2_ at 37°C. Before being treated, the cells were grown to 70–80% confluency.

The full-length *RIP3* sequence was synthesized and subcloned into a pcDNA3.1 vector. An empty pcDNA3.1 vector was employed as the NC. Lipofectamine 2000 (Promega, USA) was used to transfect M1830 cells with either the *RIP3* overexpression plasmid or the NC plasmid, in accordance with the manufacturer’s protocol. Subsequently, normal M1830 cells, M1830-*RIP3* cells and M1830-NC cells were treated with GSK’872 (5 μM) or SP600125 (10 μM).

For the LPS experiments, LPS was dissolved in pyrogen-free saline and applied at a dose of 1 μM. The cells were stimulated with LPS for four hours before being treated with GSK’872 or SP600125.

### Statistical analysis

To ensure the authenticity and reliability of the results, all biochemical indexes and tissues were acquired from ipsilateral parts (from the surgical side). Data are shown as the mean and standard deviation of at least three experiments. Differences among the groups were detected using one-way analysis of variance, and individual comparisons were made using Tukey’s *post hoc* test. IBM SPSS software version 20.0 (IBM, Armonk, NY, USA) and GraphPad Prism software version 5 (GraphPad Software, Inc., San Diego, CA, USA) were used for all statistical analyses. *P* < 0.05 was considered significant statistically.

## Supplementary Materials

Supplementary Figure
