# Supplementary material for: RIP3 Inhibition ameliorates chronic constriction injury-induced neuropathic pain by suppressing JNK signaling
Source: Aging (Albany NY). 2021 Nov 12;13(21):24417–31. doi: 10.18632/aging.203691 (PMC8610111; doi:10.18632/aging.203691)
Supplement: Supplementary Figure [file aging-13-203691-s001.pdf]

SUPPLEMENTARY FIGURE

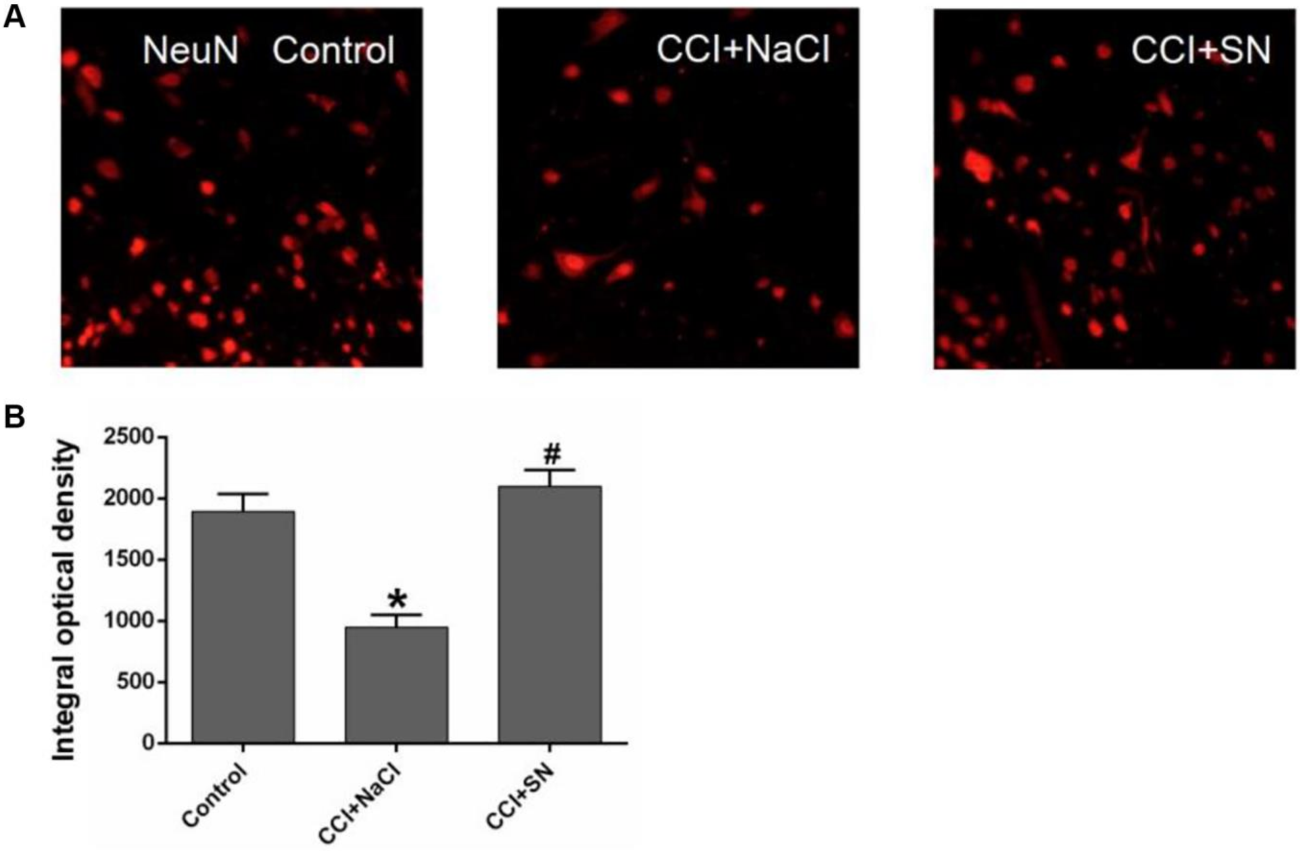

Supplementary Figure 1. SN alleviate CCI-induced neuronal death. (**A**) The expression of NeuN in lumbar spinal cord of rats treated with sinomenine by immunofluorescence on 14th day after operation. (**B**) Quantification of immunofluorescence area of (**A**) with Image J. Data are shown as mean SD (*n* = 8). $^{*}P < 0.05$, contrasted to the CCI group.
